# mRNA and miRNA Profiles of Exosomes from Cultured Tumor Cells Reveal Biomarkers Specific for HPV16-Positive and HPV16-Negative Head and Neck Cancer

**DOI:** 10.3390/ijms21228570

**Published:** 2020-11-13

**Authors:** Sonja Ludwig, Priyanka Sharma, Petra Wise, Richard Sposto, Deborah Hollingshead, Janette Lamb, Stephan Lang, Muller Fabbri, Theresa L. Whiteside

**Affiliations:** 1 Department of Otorhinolaryngology Head and Neck Surgery, University Hospital Mannheim, University of Heidelberg, 68167 Mannheim, Germany; sonja.ludwig@umm.de; 2 Department of Pathology, University of Pittsburgh School of Medicine and University of Pittsburgh Cancer Institute, Pittsburgh, PA 15213, USA; priyahpu@gmail.com; 3 Department of Pediatrics, Children′s Center for Cancer and Blood Diseases and Divisions of Hematology, Oncology, Blood and Marrow Transplantation, Children’s Hospital Los Angeles, University of Southern California, Los Angeles, CA 90027, USA; pwise@chla.usc.edu (P.W.); sposto@usc.edu (R.S.); 4 Genomics Research Core, University of Pittsburgh School of the Health Sciences, Pittsburgh, PA 15213, USA; hollings@pitt.edu (D.H.); jal18@pitt.edu (J.L.); 5 Department of Otorhinolaryngology and Head and Neck Surgery, University Hospital Essen, 45147 Essen, Germany; stephan.lang@uk-essen.de; 6 Cancer Biology Program, University of Hawai’i Cancer Center, University of Hawai’i at Manoa, Honolulu, HI 96813, USA; MFabbri@cc.hawaii.edu

**Keywords:** exosomes, head and neck cancer, HPV(+), HPV(−) tumor cells, mRNA, miR

## Abstract

Human papillomavirus (HPV)(+) and HPV(−) head and neck cancer (HNC) cells’ interactions with the host immune system are poorly understood. Recently, we identified molecular and functional differences in exosomes produced by HPV(+) vs. HPV(−) cells, suggesting that genetic cargos of exosomes might identify novel biomarkers in HPV-related HNCs. Exosomes were isolated by size exclusion chromatography from supernatants of three HPV(+) and two HPV(−) HNC cell lines. Paired cell lysates and exosomes were analyzed for messenger RNA (mRNA) by qRT-PCR and microRNA (miR) contents by nanostring analysis. The mRNA profiles of HPV(+) vs. HPV(−) cells were distinct, with *EGFR*, *TP53* and *HSPA1A/B* overexpressed in HPV(+) cells and *IL6*, *FAS* and *DPP4* in HPV(−) cells. The mRNA profiles of HPV(+) or HPV(−) exosomes resembled the cargo of their parent cells. miR expression profiles in cell lysates identified 8 miRs expressed in HPV(−) cells vs. 14 miRs in HPV(+) cells. miR-205-5p was exclusively expressed in HPV(+) exosomes, and miR-1972 was only detected in HPV(−) exosomes. We showed that HPV(+) and HPV(−) exosomes recapitulated the mRNA expression profiles of their parent cells. Expression of miRs was dependent on the HPV status, and miR-205-5p in HPV(+) and miR-1972 in HPV(−) exosomes emerge as potential discriminating HPV-associated biomarkers.

## 1. Introduction

Head and neck cancers (HNCs) belong to the ten most common malignant diseases worldwide [1]. Due to their distinct pathogenesis, HNCs are heterogeneous, and symptoms often appear when tumors are in late stages. For many years, risk factors for HNCs were alcohol consumption and the use of tobacco [2]. In recent decades, human papillomavirus (HPV) has emerged as an additional major risk factor in oropharynx, larynx and oral cancers. HPV infection is associated with 50–70% of oropharyngeal cancers in North America [3,4]. The HPV(+) oropharyngeal squamous cell carcinoma (OPSCC) differs from other HNCs by histopathologic, molecular and clinical characteristics. It is found in younger patients who usually do not have a history of alcohol abuse or smoking [5]. HPV(+) OPSCCs respond better to conventional therapy and have a significantly better outcome than HPV(−) cancers [6,7,8]. At the time of diagnosis, the majority of HNCs are locally advanced, and the standard therapy regimens are given to patients regardless of their HPV status. The sensitivity of HPV(+) HNCs to therapy suggests that therapy of HPV(+) patients with the same aggressive regimens used for HPV(−) patients may not be necessary [6]. The use of more targeted and less toxic therapies, e.g., immunotherapies in combination with chemotherapy, for treatment of HPV(+) patients offers an opportunity for improvement [9,10].

To date, it remains unclear why HPV(+) patients respond better to therapy and have a better prognosis than patients with HPV(−) cancers. The viral hypothesis associating the HPV positivity with a more robust anti-tumor immune response of cytotoxic T cells is probably most widely considered [11,12]. It suggests that the prolonged local infection with HPV activates anti-tumor immune responses and that tumor-rejection antigens released from the tumor treated with radiation or chemotherapy further drive these responses [13]. In addition, the underlying genomic differences seem to favor a better therapeutic outcome of HPV(+) HNCs [14,15]. For example, the *TP53* is mutated in 84% of HPV(−) and only 3% of HPV(+) HNCs [16]. However, no convincing evidence has so far emerged to fully support the viral-driven immune activation specifically targeting the tumor. The reasons for the disparity in therapeutic sensitivity of HPV(+) and HPV(−) cancers remain unclear, and the field is still searching for non-invasive biomarkers for treatment failure and early detection of recurrences.

We have recently reported that exosomes produced by HPV(+) HNC tumor cell lines or derived from plasma of HPV(+) patients are molecularly and functionally distinct from HPV(−) exosomes [17]. These data indicated that HPV(+) cancer cells can be distinguished from HPV(−) cancer cells at the level of exosomes [17].

Exosomes are a subset of small extracellular vesicles (EVs) sized at 30–150 nm, which are produced by all cells and circulate freely in all body fluids. Exosomes have a unique biogenesis: they are formed in the endocytic compartment of parent cells in multivesicular bodies (MVBs) and upon MVB fusion with the cellular membrane are released into tissue spaces [18]. The exosome molecular content mimics that of parent cells, and tumor-derived exosomes (TEX) are considered to be surrogates of tumor cells [18]. We reported that exosomes produced by HPV(+) HNC cells had a molecular profile distinct from that of HPV(−) exosomes and differentially reprogrammed the antigen processing machinery (APM) in human dendritic cells (DCs) [17]. The molecular and functional differences identified in these exosomes provided a partial explanation for differential interactions of HPV(+) vs. HPV(−) cancer cells with the host immune system [17].

Here, we used the same exosomes isolated from supernatants of the HPV(+) and HPV(−) HNC cell lines to study genomic mRNA and microRNA (miR) profiles of these vesicles. We report that the mRNA profiles of HNC cells and exosomes these cells produced were similar, although HPV(+) cells and exosomes were enriched in HPV-related transcripts. Analysis of miR profiles in cells and exosomes also indicated similarity and showed that fewer miRs were present in exosomes compared to cells. Surprisingly, only a single miR (miR 205-5p) was uniquely expressed in HPV(+) exosomes, while another miR (miR 1972) was only present in HPV(−) exosomes.

## 2. Results

Exosomes isolated from supernatants of all cell lines showed typical sizes, concentrations and characteristics as previously reported by us [17]. The HPV(−) exosomes induced impairment in dendritic cell (DC) maturation but had no detrimental effects on T-cell functions in co-incubation assays [17]. Total RNAs were extracted from lysates of all the cell lines, and their respective exosome fractions. The identical RNA volumes obtained from each cell line or exosome fraction were used for experiments. For mRNA analysis, real-time PCR was performed at the Genomics Research Core Facility, University of Pittsburgh. The miR analysis was performed by nanostring at Children’s Hospital Los Angeles (CHLA).

### 2.1. Heatmap Analysis of mRNA Expression Patterns in Exosomes and Corresponding Cell Lines

**Figure 1:**
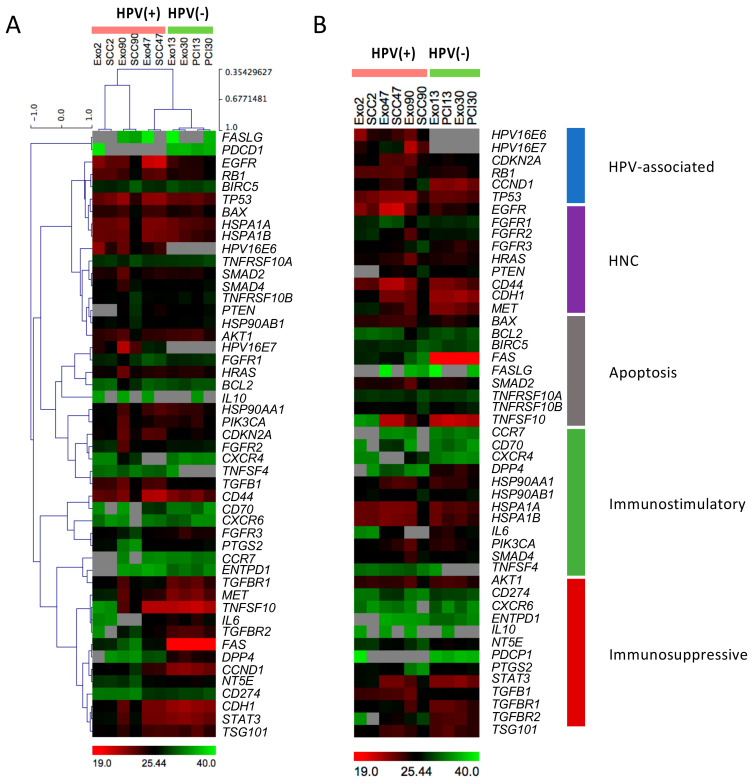
Absolute mRNA expression of HPV(+)/(−) cell lines and exosomes. mRNAs were extracted from HPV(+) and HPV(−) cell lines and corresponding exosomes isolated by size exclusion chromatography. 1 ng mRNA from cell lines and exosomes was used to detect different mRNAs by PCR and compare their Ct values. All shown absolute Ct values/1ng mRNA are means of triplicates. The heatmaps illustrate unsupervised (**A**) and supervised analysis (**B**) of the different mRNAs. Unsupervised analysis was performed using hierarchical and complete linkage clustering (Pearson correlation) to evaluate significant differences. The supervised analysis compared mRNAs for their discriminating ability in terms of the origin (HPV or head and neck cancer (HNC)) or their immunological function (apoptosis, immunostimulatory or suppressive). The color codes range from low (green, Ct value 40), through medium (black, Ct value 25.44) to high expression levels (red, Ct value 19), and not detectable (grey).

The heatmaps in Figure 1 illustrate absolute Ct values for 1 ng mRNA from each cell line or exosome fraction loaded and have been previously described [19,20,21]. Comparing absolute dCT values for 1 ng mRNA is rational since the packaging and assembly of the exosomal cargo during exosome biogenesis in late endosomes is not uniformly efficient for various cargo components. There is also no known common house-keeping gene for exosomes, as the genes commonly used for normalization of proteins in cell lysates are often not expressed to an equal extent in paired exosomes. Overall, HPV(+) compared to HPV(−) cell lines and the exosomes these cells produced show quite different mRNA profiles. Importantly, the exosomes resemble the mRNA cargos of their parent cells. The unsupervised heatmap analysis confirmed similar mRNA profiles among HPV(+) and HPV(−) cell lines and their exosomes (Figure 1A). Interestingly, the mRNA expression profile of HPV(+) SCC-47 was similar to the profiles of HPV(−) cell lines, which could be linked back to a mixed etiology of the developed cell line by HPV16 and tobacco consumption associated with most HPV-positive cell lines [22]. HPV(+) cell lines and exosomes showed elevated levels of transcripts for *EGFR*, *TP53* and *HSPA1A/B*. The mRNA transcripts for *IL6*, *FAS*, *DPP4* and *CCND1* were predominantly expressed in HPV(−) cell lines and their exosomes.

The supervised heatmap analysis grouped the mRNA profiles according to their functional impact (Figure 1B). As expected, the HPV-associated markers, *HPV16E6* and *HPV16E7*, were exclusively expressed in HPV(+) cell lines as well as in HPV(+) exosomes. *CDKN2A* which codes for p16 protein, commonly used as surrogate marker for HPV in clinical practice, was overexpressed in cell lines and exosomes from SCC-47 and exosomes from SCC-90. Overall, the immunostimulatory genes, including *HSP90*, *PIK3CA* and *SMAD4*, as well as immunosuppressive genes, such as *STAT3* and *TGFB*/*TGFBR,* were expressed at comparable levels and independently of the HPV status of exosomes or cells.

### 2.2. Direct Comparison of mRNA Profiles of Exosomes and Paired Cell Lysates

**Figure 2:**
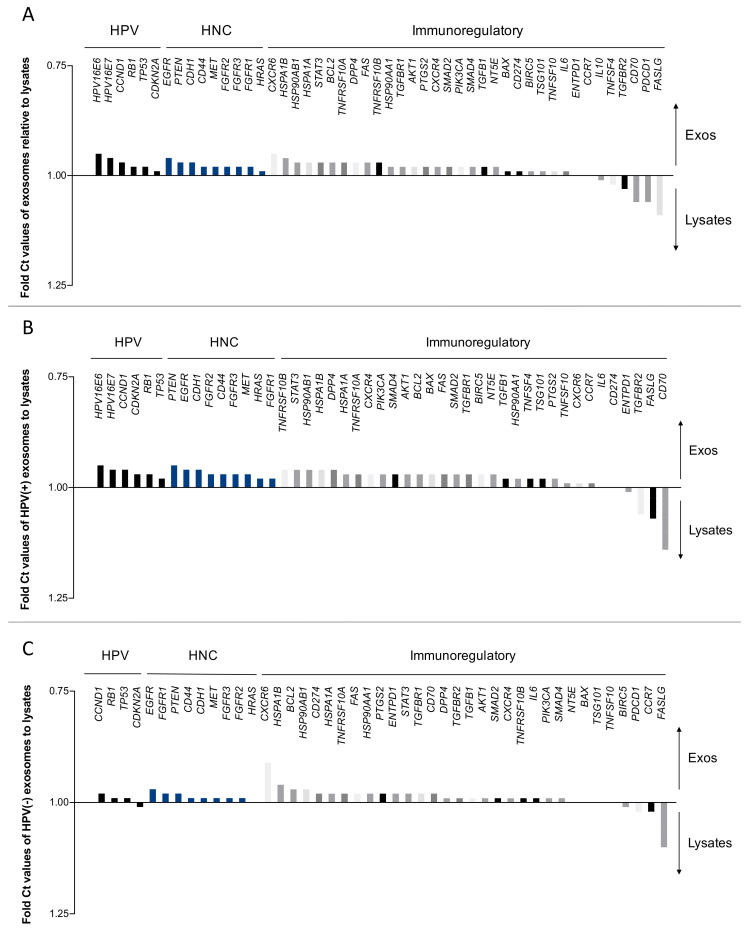
Relative mRNA expression depending on the localization in exosomes or cell lysates. Means of absolute Ct values from three replicates were compared for mRNA expression in exosomes in relation to mRNA expression in cell lysates (**A**). In the sub-analysis, relative mRNA expression was compared for HPV(+) (**B**) and HPV(−) samples (**C**). The data are presented as waterfall plots to visualize the degree of overexpression, equal expression or underexpression of mRNA in exosomes relative to the cell lysates for each mRNA species. The data are presented in fold Ct values on an inverted scale to reflect the fact that lower Ct values correspond with higher mRNA expression.

**Figure 3:**
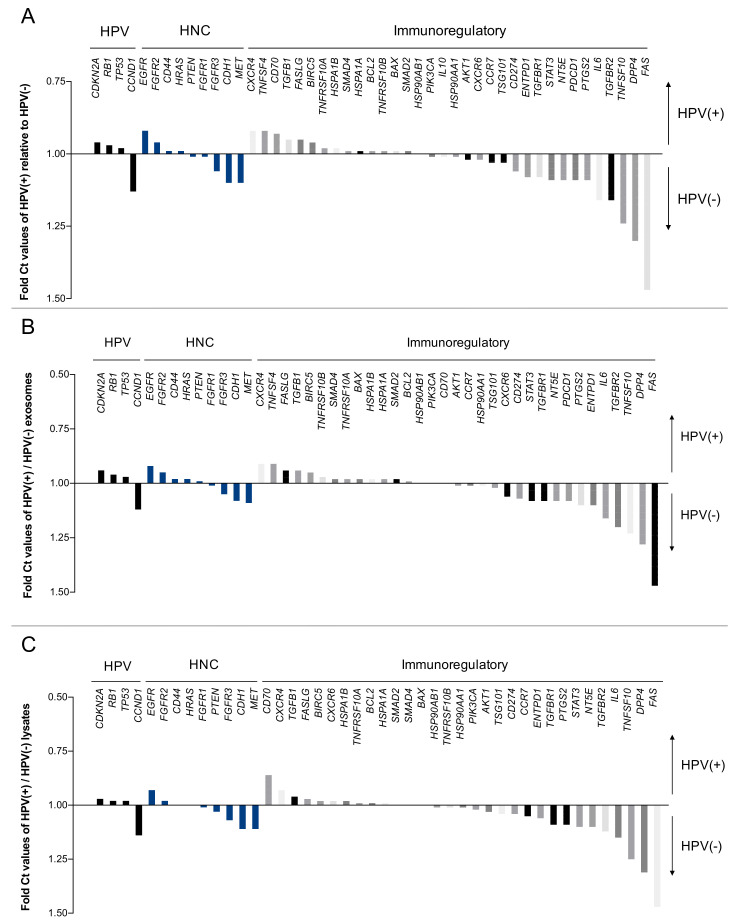
The HPV-status determines the mRNA expression profile in cell lines and exosomes. To calculate fold Ct values, means of absolute Ct values from HPV(+) samples were divided by HPV(−) results (**A**). Relative mRNA expression was examined within exosomes (**B**) and HPV cell lysates (**C**). The data are presented as waterfall plots and indicate overexpression of several immunoregulatory mRNAs within HPV(−) cell lysates and/or exosomes compared to HPV(+). The data are shown in fold Ct values on an inverted scale bar.

Waterfall plots (Figure 2 and Figure 3) were used to illustrate and directly compare each single mRNA expression for different localization (exosome vs. lysate) or the HPV-status. The mRNAs are divided into functional subgroups (as in Figure 1B). For simplicity, apoptosis, immunostimulatory and immunoinhibitory genes are combined and are presented as immunoregulatory mRNAs.

The horizontal (x) axis represents the baseline, on which both measures are equally expressed. The vertical (y) axis is used to visualize the relative amount of mRNA, which is indicated by the different vertical bars. Figure 2 compares the relative expression of each mRNA depending on the localization in exosome or the corresponding cell lysate. Strikingly, exosomes revealed higher mRNA levels of *HPV16E6* and *HPV16E7* mRNAs compared to cell lines, which could be due to the concentration of these mRNAs within the exosomes.

Expression levels of transcripts for *FASLG*, *PDCD-1* (=PD-1) and *CD70* were higher in the cell lines than in exosomes (Figure 2A). In the sub-analysis of HPV16+ cell lines, *TGFBR2*, *FASLG* and *CD70* were significantly overexpressed in cell line lysates compared to their exosomes (Figure 2B). The HPV16(−) subgroup showed overexpression of *CXCR6* in exosomes and of *FASLG* in cell lysates (Figure 2C).

The overexpression of *FASLG* and *PDCD-1* mRNAs in the cell lysates could be related to the tumor immune privilege, as is the case in many other carcinomas. By inducing apoptosis of immune cells, the cancer cells can protect themselves from immune intervention [23]. By packaging *FASLG* mRNA into exosomes, tumor cells disseminate overexpressed apoptotic signal to immune cells in the periphery [24].

### 2.3. The HPV Status Influences mRNA Profiles of Exosomes and Paired Cell Lines

In Figure 3, relative Ct values of gene expression in exosomes and cell lines are compared based on their HPV status. mRNA levels for the HPV- and HNC-associated genes *CCND1*, *CDH1* and *MET* are elevated in HPV(−) samples. Moreover, the immunoregulatory genes *TGFBR2*, *TNFSF10*, *DPP4* and *FAS* show higher values for HPV(−) than HPV(+) samples (Figure 3A). The direct comparison of HPV(+) vs. HPV(−) exosomes revealed higher levels of *CCND1*, *TNFSF10*, *DPP4* and *FAS* in HPV(−) exosomes. Overexpression of *CDKN2A*, *EGFR*, *TNFSF4* and *CXCR4* was detected in HPV(+) exosomes (Figure 3B). Similar results were obtained for the HPV-dependent comparisons of the cell lines, except for a higher expression of *CD70* in HPV(+) cells (Figure 3C).

### 2.4. The HPV Status Determines miRs Expression Levels in Exosomes and Paired Cell Lysates

Nanostring analysis identified 798 endogenous miRs in all the cell lines and respective exosomes. False discovery rate (FDR) estimation was used to identify significant differences in miR levels in cell lysates and exosomes. The data were analyzed using volcano plots, which indicated the fold differences of miRs expression levels in exosomes vs. cell lysates or the HPV status: HPV(+) vs. HPV(−).

**Figure 4:**
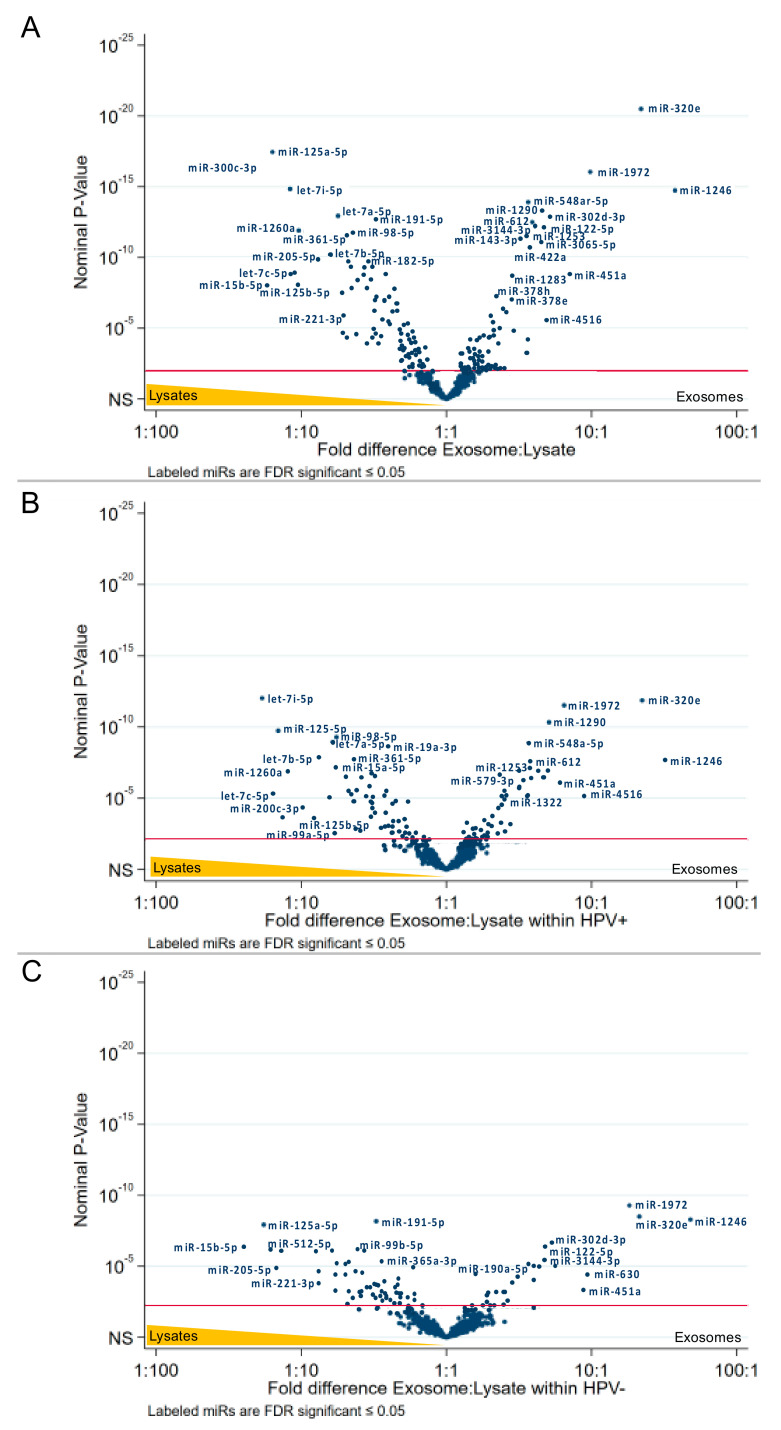
Volcano plot analysis of miRs according to their localization in exosomes and/or cell lysates. Relative expression levels of miRs are presented in fold differences exosomes:lysates as blue spots (horizontal axis). The three different graphs visualize all (**A**), HPV(+) (**B**) and HPV(−) (**C**) miRs. Dots at the right upper side are much more expressed in exosomes than in lysates, while dots at the left upper side are far more expressed in lysates than in exosomes. The vertical axis represents the significance level of the False discovery rate. *p*-value ≤ 0.05 are considered to be significant (red line).

The list of specific miRs differentially expressed with the highest significance in the analyzed subgroups is presented in Figure 4. In addition, Table S1 lists all significantly expressed miRs in detail. Several miRs are significantly differentially expressed in exosomes vs. cell lysates, and similar numbers of miRs were found to be significantly overexpressed in exosomes (80 miRs) vs. lysates (101 miRs) (Figure 4 and Table S1).

The subgroup analysis of HPV(+) cell lines revealed 69 significantly overexpressed miRs and 55 overexpressed miRs in exosomes, respectively (Figure 4A and Table S1), while the subgroup analysis of HPV(−) cell lines revealed 70 significantly overexpressed miRs and 26 overexpressed miRs in exosomes, respectively (Figure 4B,C and Table S1).

**Figure 5:**
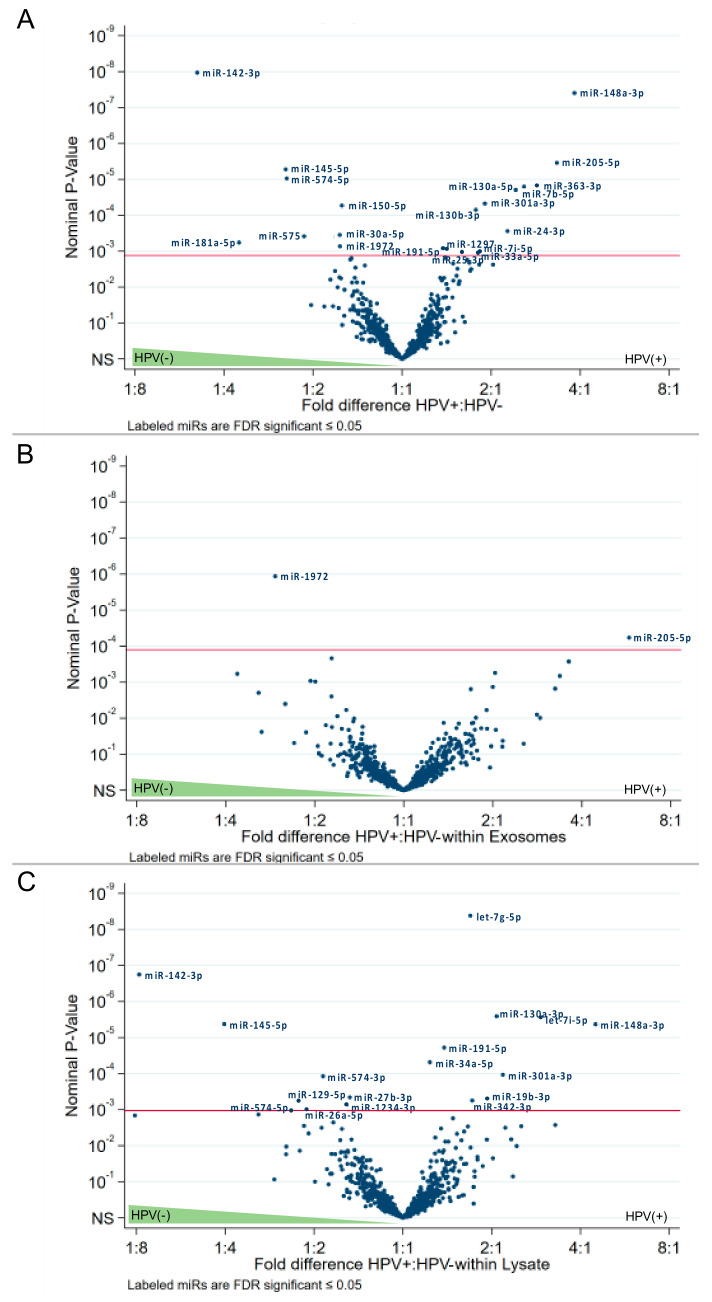
Volcano plot analysis of miRs depending on the HPV-status. Relative miR expression (blue dots) is analyzed for HPV(+) vs. HPV(−) cell lysates and exosomes (**A**), exosomes only (**B**) and lysates only (**C**). Dots at the right upper side are expressed to a higher extent in HPV(+) than in HPV(−), while dots at the left upper side are far more expressed in HPV(−) than in HPV(+). The vertical axis represents the significance level of the false discovery rate. *p*-values ≤ 0.05 are considered to be significant (red line).

Combining lysates and exosomes of HPV(+) or HPV(−) cell lines, we observed statistically significant overexpression of 8 miRs in the HPV(−) subgroup and 14 miRs in the HPV(+) subgroup (Figure 5A). Focusing only on exosomes, we observed only one miR (miR-1972) overexpressed in HPV(−) exosomes, and only one miR (miR-205-5p) overexpressed in HPV(+) exosomes (Figure 5B). However, focusing only on cell lysates, we identified nine miRs significantly overexpressed in the HPV(−) and nine different miRs in HPV(+) subgroups (Figure 5C).

**Figure 6:**
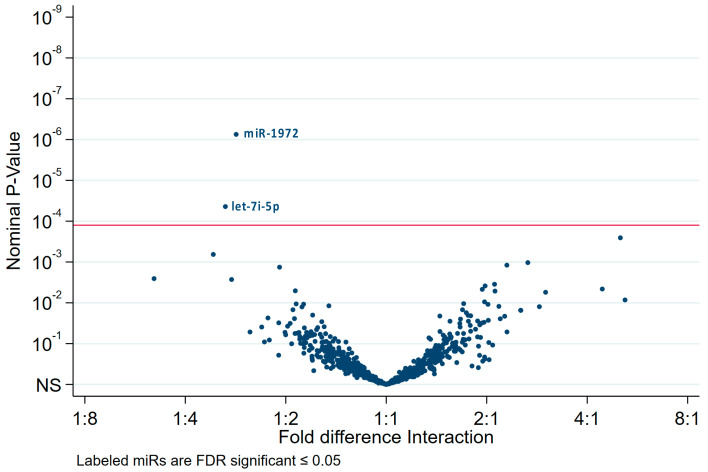
Interaction analysis of the differentially expressed miRs presented as volcano plot. The interaction analysis compared the miR expression in exosomes or cell lysates with the HPV status. Interaction analysis identified only two significant miRs: miR-1972, overexpressed in HPV(−) exosomes, and let-7i-5p, predominantly present in HPV(+) cell lysates.

The interaction analysis revealed two miRs: miR-1972, which are most likely found in HPV(−) exosomes, and let-7i-5p for HPV(+) cell lysates (Figure 6).

These results indicate that the miR cargo of HNC cell-derived exosomes differs from the miRNome of the cell that produced these exosomes. Moreover, our data indicate that HPV(+) and HPV(−) HNC cells express different signatures of miRs and release exosomes with a different miR content, suggesting that miRs in exosomes may be predictive of the HPV status of the cell that produced these exosomes.

## 3. Discussion

We and others have reported that exosomes produced by human tumor cells, TEX, exert suppressive effects on immune cells and re-program their functions [25,26,27]. Recently, we studied the molecular cargos and effects on immune cells of exosomes isolated from supernatants of HPV(+) or HPV(−) cell lines. These exosomes co-incubated with human primary T cells suppressed T-cell activation and proliferation and induced T-cell apoptosis regardless of the HPV status of parent cells [17]. However, only HPV(−) exosomes suppressed DC maturation and expression levels of proteins involved in antigen processing machinery (APM) in DCs, while HPV(+) exosomes promoted DC maturation and did not suppress APM component expression in DCs [17]. The observed disparate effects of HPV(+) vs. HPV(−) exosomes on re-programming of DC functions suggested that the reprogramming messages delivered by HPV(+) or HPV(−) exosomes to recipient DCs involved different molecular or genetic mechanisms. As the profiles of immunoregulatory proteins carried by HPV(+) and HPV(−) exosomes were similar, we considered a possibility that TEX, which are more rapidly internalized by DCs and only reluctantly by T cells [28], delivered mRNA and miR transcripts to DCs, which accounted for the observed DC reprogramming [29]. We, therefore, compared the mRNA and miR profiles of HPV(+) and HPV(−) cells as well as exosomes isolated from supernatants of these cells.

We first asked whether exosomes recapitulated the mRNA and miR content of parental HPV(+) vs. HPV(−) cells. The supervised and unsupervised analyses of HPV(+) and HPV(−) heatmaps indicated that mRNA transcripts in cells were comparable to those carried in exosomes. However, considerable differences emerged between mRNA profiles of cells and exosomes based on the HPV status. Importantly, the HPV-associated markers, *HPV16E6* and *HPV16E7* were exclusively expressed in HPV(+) lines as well as in HPV(+) exosomes, confirming the ability of exosomes to discriminate the HPV(+) from HPV(−) tumors, although mRNA levels for p16 were reported to be elevated only by some HPV(+) cell lines and exosomes (Figure 1). However, in our previous study, the p16 protein was expressed in all HPV(+) cell lines examined and paired exosomes, and no expression was evident in HPV(−) cell lines and exosomes [17], which might be caused by translational regulation.

Further, mRNA transcripts for various immunoregulatory genes, including *PIK3CA* and *SMAD4*, *TGFB/TGFBR* and *STAT3*, were expressed at comparative levels in cells and exosomes independently of their HPV status. These data are in agreement with the protein and functional data reported by us previously, which did not discriminate between HPV(+) and HPV(−) exosomes with respect to their immunosuppressive effects on T lymphocytes [17]. On the other hand, mRNA levels for the HPV- and HNC-associated genes *CCND1*, *CDH1* and *MET* were found to be overexpressed in HPV(−) exosomes as were the immunoregulatory genes *TGFBR2*, *TNFSF10*, *DPP4* and *FAS* relative to their expression in HPV(+) exosomes. This suggests that HPV(−) exosomes might play a role in promoting tumor growth and in tumor protection from immune elimination, as we have previously suggested [17]. In contrast, HPV(+) exosomes might play an immunopotentiating role [17]. Overexpression of genes such as *CDKN2A*, *EGFR*, *TNFSF4* and *CXCR4* was detected in HPV(+) exosomes.

The miR contents of the HNC cell lysates used here were previously characterized by Wald et al. [30]. We have reproduced their results and have also added the analysis of miRs in the exosomes these cells produce. The analysis of miRs in exosomes did not provide the same view of the transcript distribution between exosomes and their HPV(+) or HPV(−) parent cells as that described above for mRNA transcripts. First, fewer miR transcripts were found in exosomes than in the parent cells: 69 vs. 55 miRs in HPV(+) and 70 vs. 27 miRs in HPV(−) cells vs. exosomes. The reason for this discrepancy, which is especially evident in the HPV(−) exosomes, is not clear. It could be attributed to the less efficient packaging of exosomes in the HPV(−) than in HPV(+) cells. Interestingly, when the miRs expression profiles were compared in HPV(+) and HPV(−) cell lysates, 8 miRs were present only in HPV(−) cells and 14 only in HPV(+) cells. However, only one miR (miR-205-5p) was uniquely expressed in HPV(+) exosomes, while another miR, miR-1972, was only detected in HPV(−) exosomes. The data suggest that although exosomes carry a limited number of miRs relative to their parent cells, they can still discriminate their HPV(+) vs. HPV(−) cellular origins. Further, unique overexpression of miR-1972 in HPV(−) exosomes and of miR-205-5p in HPV(+) exosomes is especially intriguing. While the mechanistic role of differentially expressed HNC-derived exosomal miRs is beyond the scope of the present study, we noticed that miR-1972 is predicted to target the 3′-UTR region of *TAP1* [31], suggesting a possible functional role of this released miR in the alteration of the antigen processing machinery in surrounding antigen-presenting cells. The implications of such targeting might be profound for the immune response within the tumor microenvironment, and future experiments are being performed to address this point.

Overall, our genomic analysis of exosomes produced by HNC cell lines indicates that exosomes can discriminate the HPV(+) from HPV(−) cells. These results are in agreement with the proteomic and functional data we previously reported using the same HPV(+) and HPV(−) cell lines [17]. Further, our data taken together with molecular and functional analyses of the same exosomes indicate that exosomes can deliver specific miRs, such as miR-1972, to recipient DCs and thus reprogram functions of these recipient cells in favor of more effective anti-tumor immune responses, as previously suggested [32].

## 4. Materials and Methods

### 4.1. HPV-Positive and HPV-Negative Tumor Cell Lines

HPV(+) cell lines (UD-SCC-2, UM-SCC47, UPCI:SCC90) were obtained from Dr. Robert L. Ferris, HCC), and HPV(−) cell lines (PCI-13 and PCI-30) were established in our laboratories as previously reported [33]. The tumor cell lines were grown in 25 mL DMEM supplemented with 10% (*v*/*v*) exosome-depleted and heat-inactivated fetal bovine serum (Gibco, Fisher Scientific, Pittsburgh, PA, USA) and 1% (*v*/*v*) penicillin/streptomycin in 150cm^2^ flasks. After 48–72 h, cells were expanded from 40% to 80% confluency and supernatants were collected for further exosome isolation.

### 4.2. Exosome Isolation from Supernatants

Freshly harvested culture supernatants were purified by differential centrifugation at 2000× *g* for 10 min at room temperature and at 14,000× *g* for 30 min at 4 °C, and ultrafiltrated by 0.22 µm syringe-filters (Millipore, Burlington, MA, USA). Pre-clarified supernatants were concentrated 50-fold using Vivacell 100 filters (MWCO 100,000, Sartorius, Göttingen, Germany). Aliquots (1 mL) of clarified, concentrated culture supernatant were loaded on mini-SEC columns [34] and eluted with PBS to collect 1 mL of exosome fractions #3 and #4 with the highest and purest exosome content [34].

### 4.3. Total RNA Extraction

The mini SEC fractions #3 and #4 were ultracentrifuged at 100,000× *g* for 2 h, and the pelleted exosomes or HNC cells were resuspended in 350 µL of lysis buffer from the Exiqon RNA isolation kit. Total RNA was extracted following the manufacturer’s instructions. Quantity and quality of total RNA content was assessed using Nanodrop. RNA samples were aliquoted and sent to Children’s Hospital Los Angeles (CHLA) for miR analysis and to the genomics facility at the University of Pittsburgh for mRNA assessment.

### 4.4. mRNA Analysis

The exact amount of total RNA was determined using Qubit Fluorometric quantitation (Thermo Fisher Scientific, Pittsburgh, PA, USA). Reverse transcription was performed on 100 ng of total RNA in triplicate using QuantiTect Reverse Transcription Kit (Qiagen, Hilden, Germany). PCR was performed with cDNA of 1 ng RNA equivalence per well. All real-time PCRs were run on Applied Biosystems 7900HT (Applied Biosystems, Foster, CA, USA). Newly designed primers for E6 and E7 (Table S2) were validated with artificial gBlock template as well as the known positive control. Primer efficiency was verified to be consistent over 4 orders of magnitude. These assays and Hsp90AA1 and HSP90Ab1 were set up with 2 ng cDNA equivalence in a 10 μL reaction in a 384-well plate. Absolute dCt values of the cell lysates and exosomes were visualized by heatmaps using the Multiple experiment viewer software (MEV, version 4, http://mev.tm4.org). Relative dCt values were calculated for all exosome specimens in relation to their individual cell line or in relation to the HPV-status.

### 4.5. miR Analysis and Data Interpretation

The data comprised results of miR count values from nanostring assays for exosomes and cell lysates from five cell lines, two of which were HPV(−) (PCI-13 and PCI-30) and three HPV(+) (SCC-2, SCC-47 and SCC-90). Three replicate assays were performed for each cell line for both lysate and exosomes.

The nanostring assay identified 828 miRs. Of these, 798 were classified as endogenous, 5 housekeeping, 6 ligations, 8 negative controls, 6 positive controls, and 5 spike-ins. This analysis was restricted to endogenous miRs.

Endogenous miR count values were transformed to the log10 scale and then quantile-normalized along with data from six assays from an unrelated HUVEX experiment [35]. For each miR, individual ordinary least squares regression analyses of normalized counts on sample type (exosome vs. lysate) and HPV status (HPV(+) vs. HPV(−)) were performed. The statistical significance of the difference in expression between exosomes and lysates and between HPV(+) and HPV(−) cell lines was assessed in the context of this main effect model. Implicit in this analysis is the assumption that differences in expression between HPV(+) and HPV(−) cell lines are similar in both exosomes and cell lysates and, conversely, that differences in expression between exosomes and cell lysates are similar in both HPV(+) and HPV(−) cell lines. In addition, regression analysis on sample type within HPV status and on HPV status within sample type was performed. In addition, regression analysis on sample type and HPV status that included both main effects and an interaction term was performed to test formally whether differences due to HPV status differed by sample type and vice versa. Within each analysis, overall Type I error was controlled by declaring individual miRs analyses significant if they satisfied an FDR level of 0.05 according to the Benjamini and Hochberg false discovery rate (FDR) adjustment [36]. The data were visualized by volcano plots. The abscissa is the estimated difference in quantiles normalized log10 count of the subgroups, and the ordinate is the nominal *p*-value (log10 scale). Note that “hsa” has been removed from the miR names in the plots and Supplementary Tables for better visualization. The level of FDR significance (*p* < 0.05) is highlighted by a red line in the graphs.

## Supplementary Materials

Supplementary materials can be found at https://www.mdpi.com/1422-0067/21/22/8570/s1. Table S1: Detailed list of miR expression in cell lysates and exosomes, Table S2: Detailed list of IDT primers (A) and Qiagen primers (B) used for mRNA analysis.

## Abbreviations

APM	Antigen processing machinery of dendritic cells
CDKN2A	Cyclin-Dependent Kinase Inhibitor 2A
CXCR4	C-X-C chemokine receptor type 4
DMEM	Dulbecco’s Modified Eagle Medium
DPP4	Dipeptidyl-peptidase 4
EGFR	Epidermal growth factor receptor
FDR	False discovery rate
HNC	Head and neck cancer
HPV	Human papillomavirus
HPV(+)	HPV-positive
HPV(-)	HPV-negative
HSP	Heat shock protein
IL	Interleukin
mRNA	Messenger RNA
miR	Micro RNA
OPSCC	Oropharyngeal squamous cell carcinoma
PD-1	Programmed cell death protein 1
PD-L1	Programmed cell death-ligand 1
PIK3CA	Phosphatidylinositol-4,5-Bisphosphate 3-Kinase Catalytic Subunit Alpha
TAP1	Transporter associated with Antigen Processing 1
TEX	Tumor-derived exosomes
TGFB/TGFBR	Transforming growth factor beta/receptor
TNFSF	Tumor necrosis factor (ligand) superfamily
