# Supplementary material for: mRNA and miRNA Profiles of Exosomes from Cultured Tumor Cells Reveal Biomarkers Specific for HPV16-Positive and HPV16-Negative Head and Neck Cancer
Source: Int J Mol Sci. 2020 Nov 13;21(22):8570. doi: 10.3390/ijms21228570 (PMC7698015; doi:10.3390/ijms21228570)
Supplement: Supplementary file 1 [file ijms-21-08570-s001.pdf]

Supplementary Table 1: Detailed list of miR expression in cell lysates and exosomes

The following tables specify all miRs significantly overexpressed in lysates or exosomes (A), exclusively for HPV(+) (B) and for HPV(-) only (C). A significance level in FDR test with a p-value <0.05 was considered as significant.

A. miR expression in HPV(+) and HPV(-) lysates vs. exosomes

Lysates	Exosomes
miR-15b-5p	miR-3605-5p
miR-125a-5p	miR-885-5p
let-7i-5p	miR-487b-3p
let-7c-5p	miR-524-3p
miR-200c-3p	miR-1249-3p
miR-125b-5p	miR-548ar-3p
miR-1260a	miR-887-5p
miR-205-5p	miR-190a-3p
let-7b-5p	miR-587
let-7a-5p	miR-1295a
let-7d-5p	miR-548e-5p
miR-221-3p	miR-378d
miR-181a-5p	miR-133a-5p
miR-361-5p	miR-517c-3p
miR-99a-5p	miR-519a-3p
let-7e-5p	miR-494-5p
miR-15a-5p	miR-548a-5p
miR-98-5p	miR-564
miR-31-5p	miR-1286
miR-4443	miR-1302
miR-29b-3p	miR-370-3p
miR-331-3p	miR-411-5p
miR-423-3p	miR-604
let-7g-5p	miR-1289
miR-200b-3p	miR-520h
miR-182-5p	miR-378f
miR-196b-5p	miR-376c-5p
miR-324-5p	miR-508-5p
miR-4454	miR-33b-5p
miR-7975	miR-607
miR-99b-5p	miR-1303
let-7f-5p	miR-548ad-3p
miR-92a-3p	miR-933
miR-19b-3p	miR-199a-3p
miR-93-5p	miR-199b-3p
miR-23b-3p	miR-495-3p
miR-191-5p	miR-4647
miR-135b-5p	miR-548y
miR-106a-5p	miR-514b-5p
miR-17-5p	miR-579-3p
miR-374a-5p	miR-563
miR-151a-3p	miR-378i
miR-151a-5p	miR-543
miR-9-5p	miR-520b
miR-1180-3p	miR-1281
miR-92a-1-5p	miR-548g-3p
miR-29a-3p	miR-664b-3p

miR-19a-3p<br>miR-361-3p<br>miR-100-5p<br>miR-27b-3p<br>miR-30b-5p<br>miR-345-5p<br>miR-106b-5p<br>miR-193a-5p<br>miR-193b-5<br>miR-107<br>miR-29c-3p<br>miR-574-3p<br>miR-197-3<br>miR-181b-5p<br>miR-181d-5p<br>miR-425-5p<br>miR-365a-3p<br>miR-365b-3p<br>miR-503-5p<br>miR-96-5p<br>miR-484<br>miR-539-5p<br>miR-363-3p<br>miR-374b-5p<br>miR-92b-3p<br>miR-342-3p<br>miR-532-5p<br>miR-504-5p<br>miR-26a-5p<br>miR-20a-5p<br>miR-20b-5p<br>miR-1306-5p<br>miR-671-3p<br>miR-6724-5p<br>miR-30c-5p<br>miR-767-3p<br>miR-30d-5p<br>miR-105-5p<br>miR-28-5p<br>miR-34a-5p<br>miR-551a<br>miR-30a-3p<br>miR-28-3p<br>miR-194-5p<br>miR-500a-5p<br>miR-501-5p<br>miR-193b-3p<br>miR-4741<br>miR-195-5p<br>miR-130b-3p<br>miR-1234-3p<br>miR-5196-3p<br>miR-6732-3p<br>miR-185-5p	miR-1285-5p<br>miR-502-5p<br>miR-548ah-5p<br>miR-155-5p<br>miR-888-5p<br>miR-2682-5p<br>miR-1276<br>miR-1322<br>miR-1305<br>miR-378h<br>miR-575<br>miR-128-3p<br>miR-585-3p<br>miR-494-3p<br>miR-378e<br>miR-1283<br>miR-144-3p<br>miR-143-3p<br>miR-1253<br>miR-630<br>miR-548ar-5p<br>miR-422a<br>miR-612<br>miR-3144-3p<br>miR-3065-5p<br>miR-1290<br>miR-122-5p<br>miR-4516<br>miR-302d-3p<br>miR-451a<br>miR-1972<br>miR-320e<br>miR-1246

B. miR expression in HPV(+) lysates vs. exosomes

HPV (+) Lysates	HPV(+) Exosomes
let-7i-5p	miR-1249-3p
let-7c-5p	miR-130a-3p
miR-125a-5p	miR-548e-5p
miR-15b-5p	miR-411-5p
miR-1260a	miR-328-5p
miR-200c-3p	miR-133a-5p
miR-125b-5p	miR-345-3p
let-7b-5p	miR-1295a
let-7d-5p	miR-548a-5p
let-7a-5p	miR-1286
miR-99a-5p	miR-376c-5p
miR-98-5p	miR-548y
miR-15a-5p	miR-1303
miR-205-5p	miR-888-5p
miR-4454	miR-607
miR-7975	miR-4647
let-7e-5p	miR-582-5p
miR-361-5p	miR-1281
miR-31-5p	miR-199a-3p
miR-29b-3p	miR-199b-3p
miR-4443	miR-604
miR-221-3p	miR-933
miR-182-5p	miR-525-5p
miR-196b-5p	miR-1276
let-7g-5p	miR-563
miR-324-5p	miR-502-5p
miR-151a-3p	miR-1285-5p
miR-19b-3p	miR-520b
miR-331-3p	miR-585-3p
miR-23b-3p	miR-579-3p
let-7f-5p	miR-144-3p
miR-92a-1-5p	miR-2682-5p
miR-151a-5p	miR-378h
miR-191-5p	miR-128-3p
miR-374a-5p	miR-1322
miR-99b-5p	miR-155-5p
miR-423-3p	miR-1305
miR-100-5p	miR-143-3p
miR-30b-5p	miR-494-3p
miR-19a-3p	miR-1283
miR-493-3p	miR-378e
miR-107	miR-548ar-5p
miR-539-5p	miR-1253
miR-361-3p	miR-3144-3p
miR-345-5p	miR-612
miR-106b-5p	miR-3065-5p
miR-1180-3p	miR-422a
miR-425-5p	miR-122-5p
miR-342-3p	miR-302d-3p
miR-92a-3p	miR-1290
miR-96-5p	miR-451a
miR-195-5p	miR-1972
miR-491-5p	miR-4516
miR-374b-5p	miR-320e
miR-30d-5p	miR-1246

miR-92b-3p<br>miR-29c-3p<br>miR-6724-5p<br>miR-29a-3p<br>miR-671-3p<br>miR-532-5p<br>miR-125a-3p<br>miR-409-3p<br>miR-503-5p<br>miR-936<br>miR-193b-3p<br>miR-1306-5p<br>miR-30e-3p<br>miR-873-3p	

C. miR expression in HPV(-) lysates vs. exosomes

HPV(-) Lysates	HPV(-) Exosomes
miR-15b-5p	miR-190a-5p
miR-125a-5p	miR-548g-3p
miR-125b-5p	miR-378e
miR-205-5p	miR-1268a
miR-181a-5p	miR-514b-5p
miR-200c-3p	miR-1283
miR-1260a	miR-1276
miR-221-3p	miR-664b-3p
let-7c-5p	miR-585-3p
let-7i-5p	miR-888-5p
miR-361-5p	miR-422a
miR-92a-3p	miR-1253
miR-423-3p	miR-143-3p
miR-200b-3p	miR-548ar-5p
let-7e-5p	miR-1290
let-7a-5p	miR-612
miR-31-5p	miR-575
miR-93-5p	miR-3144-3p
miR-296-5p	miR-122-5p
miR-331-3p	miR-302d-3p
let-7b-5p	miR-3065-5p
miR-135b-5p	miR-451a
miR-106a-5p	miR-630
miR-17-5p	miR-1972
miR-4443	miR-320e
miR-99b-5p	miR-1246
miR-29a-3p	
let-7g-5p	
let-7d-5p	
miR-9-5p	
miR-99a-5p	
miR-193a-5p	
miR-193b-5p	
miR-15a-5p	
miR-324-5p	
let-7f-5p	
miR-98-5p	
miR-27b-3p	
miR-191-5p	
miR-196b-5p	

miR-19b-3p<br>miR-1180-3p<br>miR-574-3p<br>miR-182-5p<br>miR-23b-3p<br>miR-365a-3p<br>miR-365b-3p<br>miR-374a-5p<br>miR-24-3p<br>miR-145-5p<br>miR-181b-5p<br>miR-181d-5p<br>miR-29c-3p<br>miR-197-3p<br>miR-503-5p<br>miR-129-5p<br>miR-504-5p<br>miR-30a-3p<br>miR-484<br>miR-361-3p<br>miR-151a-5p<br>miR-133b<br>miR-1306-5p<br>miR-151a-3p<br>miR-105-5p<br>miR-34a-5p<br>miR-1234-3p<br>miR-4524a-5p<br>miR-23a-3p<br>miR-1910-3p	

Supplementary Table 2. Detailed list of IDT primers (A) and Qiagen primers (B) used for mRNA analysis

A. IDT primers

**Gene Symbol**	**Forward primer (5′-3′)**	**Reverse primer (3′-5′)**
HPV16E6	AATGTTTCAGGACCCACAGG	CCCGAAAAGCAAAGTCATATACC
HPV16E7	CGGACAGAGCCCATTACAAT	TCTTCCAAAGTACGAATGTCTACG

B. Qiagen primers

**Gene Symbol**	**Refseq #**	**Official Full Name**	**Qiagen catalog Number**
CCND1	NM_053056	cyclin D1	PPH00128F
TP53	NM_000546	tumor protein p53	PPH00213F
PTEN	NM_000314	phosphatase and tensin homolog	PPH00327F
RB1	NM_000321	retinoblastoma 1	PPH00228F
EGFR	NM_005228	epidermal growth factor receptor	PPH00138B

FGFR1	NM_000604	fibroblast growth factor receptor 1	PPH00372F
FGFR2	NM_000141	fibroblast growth factor receptor 2	PPH00391F
FGFR3	NM_000142	fibroblast growth factor receptor 3	PPH00382A
CD44	NM_000610, NM_001001389, NM_001001390, NM_001001391, NM_001001392, NM_001202555, NM_001202556, NM_001202557	CD44 molecule (Indian blood group)	PPH00114A
CDH1	NM_004360	cadherin 1, type 1, E-cadherin (epithelial)	PPH00135F
MET	NM_000245	MET proto-oncogene, receptor tyrosine kinase	PPH00194A
HRAS	NM_001130442	Harvey rat sarcoma viral oncogene homolog	PPH00159C
PDCD1	NM_005018	programmed cell death 1	PPH13086G
CD274	NM_014143	CD274 molecule	PPH21094A
FAS	NM_000043	Fas cell surface death receptor	PPH00141B
FASLG	NM_000639	Fas ligand (TNF superfamily, member 6)	PPH00142C
PTGS2	NM_000963	prostaglandin-endoperoxide synthase 2 (prostaglandin G/H synthase and cyclooxygenase)	PPH01136F
CD70	NM_001252	CD70 molecule	PPH00258F
TNFSF4	NM_003326	tumor necrosis factor (ligand) superfamily, member 4	PPH00820D
TGFB1	NM_000660	transforming growth factor, beta 1	PPH00508A
TGFBR1	NM_001130916	transforming growth factor, beta receptor 1	PPH00237C
TGFBR2	NM_001024847	transforming growth factor, beta receptor II (70/80kDa)	PPH00339C
IL10	NM_000572	interleukin 10	PPH00572C
ENTPD1	NM_001098175	ectonucleoside triphosphate diphosphohydrolase 1	PPH21158A
NT5E	NM_001204813	5'-nucleotidase, ecto (CD73)	PPH12908E
TNFSF10	NM_001190942	tumor necrosis factor (ligand) superfamily, member 10	PPH00242F

TNFRSF10A	NM_003844	tumor necrosis factor receptor superfamily, member 10a	PPH00842A
TNFRSF10B	NM_003842	tumor necrosis factor receptor superfamily, member 10b	PPH00241C
SMAD2	NM_001003652	SMAD family member 2	PPH01949F
SMAD4	NM_005359	SMAD family member 4	PPH00134C
STAT3	NM_003150	signal transducer and activator of transcription 3 (acute-phase response factor)	PPH00708F
BCL2	NM_000633, NM_000657	B-cell CLL/lymphoma 2	PPH00079B
BIRC5	NM_001012270	baculoviral IAP repeat containing 5	PPH00271E
BAX	NM_004324, NM_138761, NM_138763, NM_138764, NM_138765, NR_027882	BCL2-associated X protein	PPH00078B
PIK3CA	NM_006218	phosphatidylinositol-4,5-bisphosphate 3-kinase, catalytic subunit alpha	PPH01355A
AKT1	NM_001014431, NM_001014432, NM_005163	v-akt murine thymoma viral oncogene homolog 1	PPH00088B
TSG101	NM_006292	tumor susceptibility 101	PPH06937E
HSPA1A	NM_005345	heat shock 70kDa protein 1A	PPH01193B
HSPA1B	NM_005346	heat shock 70kDa protein 1B	PPH01216B
IL6	NM_000600	interleukin 6	PPH00560C
CCR7	NM_001838	chemokine (C-C motif) receptor 7	PPH00617A
DPP4	NM_001935	dipeptidyl-peptidase 4	PPH00035B
CXCR4	NM_001008540	chemokine (C-X-C motif) receptor 4	PPH00621A
CXCR6	NM_006564	chemokine (C-X-C motif) receptor 6	PPH01343A
CDKN2A	NM_000077	cyclin-dependent kinase inhibitor 2A	PPH00207C
